# Enhancing Quality and Processing Efficiency of Germinated Buckwheat Tea Through Hot Air-Assisted Radio Frequency Roasting

**DOI:** 10.3390/foods14213596

**Published:** 2025-10-22

**Authors:** Hsiang-Yu Lai, Jui-Min Hsiao, Su-Der Chen

**Affiliations:** 1 Department of Food Science, National Ilan University, Number 1, Section 1, Shen-Lung Road, Yilan City 260007, Taiwan; r1232009@niu.edu.tw; 2 Department of Applied Economics and Management, National Ilan University, Number 1, Section 1, Shen-Lung Road, Yilan City 260007, Taiwan; jmhsiao@niu.edu.tw

**Keywords:** germinated buckwheat, radio frequency roasting, aroma profile, sensory evaluation, energy efficiency

## Abstract

Buckwheat germination can increase bioactive compounds; however, it also increases moisture content, which then requires drying and roasting. This study focused on applying hot air-assisted radio frequency (HARF) roasting to germinated buckwheat (GB) tea to improve the tea quality and processing efficiency. Seeds were soaked in reverse osmosis water for 6 h, followed by germination at 25 °C for 24 h. HARF roasting (100 °C, 10 kW, 16 cm gap) rapidly heated one bucket (2 kg) and two buckets (2 + 2 kg) of GB to 140 °C in only 22 and 20 min, respectively, to reduce the moisture content from 40% to 5%. HARF roasting could save about 1/5 time and 1/11~1/18 energy compared with a cyclonic oven at 140 °C for 100 min roasting to 120 °C. GC-IMS and sensory evaluation indicated a superior aroma, taste, and higher overall acceptability for HARF-roasted GB tea. These results demonstrate that GB using HARF roasting enhances the functional, sensory, and industrial value of buckwheat tea.

## 1. Introduction

Buckwheat (*Fagopyrum* spp.) is a nutritional pseudo-cereal with several health benefits, being notably rich in rutin, quercetin, and other polyphenols that exhibit significant antioxidant, anti-inflammatory, and cardioprotective effects [1]. Germinated buckwheat (GB) has shown ~20–86% higher total polyphenols and improves 2,2-diphenyl-1-picrylhydrazyl (DPPH) radical scavenging capacity; therefore, germination further elevates its bioactive constituents to increase antioxidant activity [2].

High-moisture GB must be dried and roasted to produce low-moisture tea with a pleasant aroma. Conventional convective (cyclone) roasting involves long heating times and low thermal efficiency, which may cause nutrient loss and reduce flavor quality. In contrast, radio frequency (RF) heating provides rapid, uniform, and volumetric heating, making it a promising technology for food processing [3]. RF can improve thermal efficiency while maintaining nutritional and sensory quality; for instance, Ling et al. [4] used a 10 kW RF system to rapidly heat grains to target temperatures, significantly preserving antioxidants and increasing overall thermal efficiency—features suitable for industrial scale-up. A 10 kW RF direct roasting process for GB, implemented with a continuous conveyor rather than separate RF drying and RF roasting stages, can further enhance the downstream thermal efficiency and quality consistency; this combined approach provides a robust technological basis and application outlook for industrializing high-quality, health-oriented buckwheat tea.

Gas chromatography–ion mobility spectrometry (GC-IMS), which couples GC’s separation power with IMS’s high sensitivity at ambient pressure, is suitable for the rapid detection of volatiles; its 2D “fingerprint” plots intuitively display compositional patterns under different processing conditions [5]. Beyond bitter buckwheat flavor studies, GC-IMS is widely applied to cereals [6] and meats [7] for quality assessment and origin authentication. Moreover, Parastar and Weller [8] noted GC-IMS to be more resource-efficient and environmentally friendly than GC-MS, making it a “green” alternative for gas-phase analysis.

Therefore, this study aimed to produce GB tea via 10 kW, 40.68 MHz hot air-assisted radio frequency (HARF) direct roasting, and to evaluate infusion quality using a nine-point hedonic test and GC-IMS aroma analysis, with the goals of improving product quality and processing efficiency.

## 2. Materials and Methods

### 2.1. Materials

Commercial dried buckwheat (Taichung no. 2 bitter buckwheat, 2024 crop) was purchased from “Mai Fashion” in Changhua, Taiwan (2nd cropping; harvested 28 Jan 2024) and stored at 4 °C. The commercial buckwheat tea was purchased from Kuang Chuan Foods (Taipei, Taiwan; lot no. T0924), produced from roasted unhulled Tartary buckwheat and vacuum-packed within three months.

### 2.2. Preparation of GB Tea

Following Tsou et al. [9] with modifications, impurities were removed, and 500 g of buckwheat was soaked in distilled water (PP box; grain/water 1:2, *w*/*w*) at 25 °C for 6 h. After draining, seeds were spread in a ventilated basket and germinated at 25 °C in the dark to obtain GB after 24 h.

### 2.3. HARF Roasting of GB Tea

A HARF unit (EDC-10D, max output 10 kW, 40.68 MHz, 220 V; I-Da Biotech, Yilan, Taiwan) with a 65 cm × 45 cm electrode received 100 °C forced air (2.5 m/s) from the rear. Loads of 1, 1.5, and 2 kg were placed in plastic baskets (Ø 29 cm, H 9.5 cm). Under various electrode gaps, ~5 s heating pulses were applied, and the output current (A) was recorded. Given the device’s maximum current (2.2 A) and power (10 kW), the average RF output power was computed as follows:

(1) Power output (kW) = (10/2.2) × A × (220/380)

where *A* is the measured output current (A). The denominator (380 V) represents the rated three-phase line voltage of the generator, which was used to normalize the measured single-phase current and convert it to equivalent power output under the 10 kW system rating.

The overall experimental workflow for the preparation, drying, roasting, and evaluation of germinated buckwheat (GB) tea is illustrated in Figure S1 (Supplementary Materials). The schematic diagram shows the sequence of soaking, germination, HARF roasting, and subsequent analyses, including color measurement, sensory evaluation, and GC–IMS aroma profiling. This workflow clarifies the relationship among each processing step and the analytical evaluations conducted in this study.

### 2.4. Temperature and Moisture Changes During HARF Drying

GB, with an initial moisture content of approximately 40% (before roasting), was heated using 10 kW, 40.68 MHz RF, and 100 °C hot air until the final moisture content decreased to about 5% (d.b., after roasting), and the product temperature reached approximately 140 °C. Single-basket and double-basket loads of 1, 1.5, and 2 kg were processed in baskets (Ø 28.8 cm, H 9.5 cm) beneath a 65 cm × 45 cm electrode with 100 °C forced air from the rear. Appropriate gaps were selected and drying/temperature curves plotted to determine optimal conditions. A handheld IR thermometer (Testo 104-IR, Dest Instrument Co., New Taipei, Taiwan) measured surface temperatures at three locations every 120 s; means of triplicate runs formed heating curves. Moisture on a dry basis (d.b.) was inferred from the mass loss during RF treatment, with the dry material ratio % determined using a 105 °C oven (Channel DCM45, KoHua, Taiwan) to constant weight.

(2) Moisture content (d.b.) = (W_t_ − W_o_)/W_o_

where W_t_ is the sample weight at time t, and W_o_ = initial weight × dry matter ratio.

### 2.5. Energy Consumption During RF Drying and Roasting

A clamp AC ammeter (3280-10, Hioki Taiwan Co., Ltd., Taipei, Taiwan) recorded three-phase current (A) every 2 min until moisture fell below 14% (total drying time defined as 120 min). The total energy consumption (E) was calculated as follows:

(3) E (kWh) = 220 × A × √3 × drying time (h)/1000

where 220 voltage (V); A = current (A); and √3 = three-phase conversion factor.

### 2.6. Cyclone Oven Roasting of GB Tea

Next, 1 kg samples were spread on trays and roasted in a preheated cyclone oven at 140 °C; samples were turned once at 20 min [10]. The current was logged every 20 min, and power/energy was calculated as follows:

(4) P (kW) = V × I/1000

(5) E (kWh) = P × t (h)

where P = power, V = 220 V, and I = A.

### 2.7. Color Analysis of GB Tea

A colorimeter (Hunter L.A.B, Color Flex, Reston, VA, USA) measured L*, a*, and b* (*n* = 6). L* indicates lightness (0 = black, 100 = white); a* red(+)–green(−); and b* yellow(+)–blue(−). The color difference Δ*E* (0–100; lower = smaller difference) was computed with control (ungerminated) values for L*, a*, and b* and experimental (germinated) values for L_1_*, a_1_*, and b_1_*:

(6) $$∆E=\sqrt{{\left(L^{*}-L_{1}^{*}\right)}^{2}+{\left(a^{*}-a_{1}^{*}\right)}^{2}+{\left(b^{*}-b_{1}^{*}\right)}^{2}}$$

### 2.8. Nine-Point Hedonic Sensory Evaluation

Following the protocol by Ryu et al. [11], 3 g roasted GB tea was infused in 120 mL water at 95 °C, boiled for 3 min, filtered, and served at ≤55–60 °C. After instructions, panelists evaluated appearance under a standardized D65 light source against a matte white background. Appearance assessment covered color intensity, clarity, and uniformity. The palate was reset with water between samples. The sample order was randomized by software; the codes had three digits. A 9-point hedonic scale (1 = dislike extremely, 5 = neither, 9 = like extremely) was used for color, smell, taste, and overall acceptability. A rank-order test (1 = most preferred) was also conducted with no duplicate codes. The sensory evaluation (*n* = 56; age 20–45 years; 32 females, 24 males) conducted in this study involved only voluntary participants (naive consumers), who tasted buckwheat tea samples prepared under food-safe conditions. No personal data, medical intervention, or vulnerable populations were involved. Therefore, ethics approval was not required. Nevertheless, all participants provided informed consent prior to the test.

### 2.9. GC-IMS Analysis of Volatiles

Following Fan et al. [5], volatiles were analyzed using a GC-IMS (FlavourSpec^®^, G.A.S., Dortmund, Germany) equipped with an OV-5 non-polar capillary column (20 m × 0.53 mm × 1 µm; 5% diphenyl–95% polydimethylsiloxane). One gram of ground sample was placed in a 20 mL headspace vial, 5 mL boiling water was added, and the vial was incubated at 60 °C for 20 min. A heated autosampler syringe (80 °C) injected 200 µL headspace gas. The GC column was held at 60 °C for a 20 min run. The carrier gas (N_2_, 99.999%) flow rate gradient was as follows: 2 mL/min for 2 min; 10 mL/min for 8 min; 50 mL/min for 10 min; and 150 mL/min for 10 min. Identification used the retention index (RI) and drift time (DT) with tolerances of ±10% and ±0.02%, respectively. Data were acquired and processed using the G.A.S. IMS Control TFTP Server (LAV software version 2.0.0).

### 2.10. Statistical Analysis

The results are expressed as mean ± SD. Statistical analyses were performed in SAS Enterprise Guide 8.3 (SAS Institute Inc., Cary, NC, USA). Group differences were tested by Duncan’s Multiple Range Test at α = 0.05. GC-IMS data were first processed in VOCal GC-IMS Measurement Evaluation Software (v0.4.8.364; G.A.S. mbH, Dortmund, Germany) for peak picking, peak area, and retention/drift time standardization, identifications referenced the built-in library, and 2D/3D plots were generated for downstream statistics. The resulting peak area matrices were autoscaled (mean-centered, unit variance), and principal component analysis (PCA) was performed directly in VOCal to evaluate differences among treatments. PC1 and PC2 were used to interpret sample separation patterns.

## 3. Results and Discussion

### 3.1. The 10 kW RF Roasting of GB Tea

**Figure 1:**
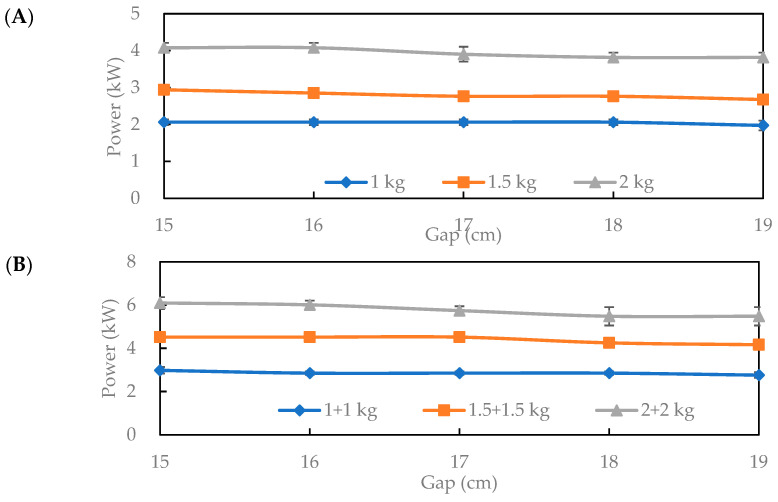
Effects of loading capacity with (**A**) a single basket, (**B**) double baskets, and gaps of GB on RF power under 100 °C hot air and 10 kW radio frequency conditions.

Hot air-assisted radio frequency (HARF) roasting has been widely applied to the rapid drying of cereals/legumes/germinated materials. The direct HARF processing of GB at about 40% moisture to 5% moisture GB tea is expected to save time. Figure 1 shows the effects of electrode gap and load on RF output power with 100 °C hot air and a 10 kW RF. With a single basket at a 16 cm gap, 2 kg achieved about 4.3 kW, which is significantly higher than 1.5 kg (2.9 kW) and 1 kg (2.1 kW). Considering continuous production with two baskets beneath the electrode, the 2 + 2 kg configuration yielded ~5.9 kW, exceeding 1.5 + 1.5 kg (4.6 kW) and 1 + 1 kg (3.2 kW). Thus, higher loads enhance RF energy absorption, consistent with increased dielectric loss and more focused internal heating as bulk volume rises [12]. Small loads or wide gaps promote reflection/loss and reduce absorption. Balancing stability and practicality, a 16 cm gap was selected for subsequent drying and roasting [13].

**Figure 2:**
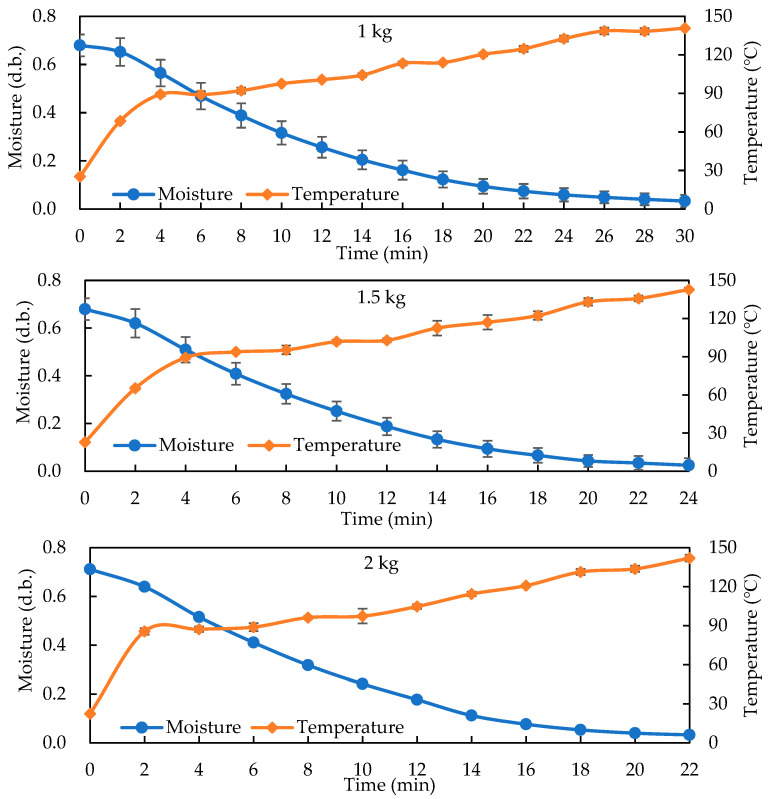
Drying curves and temperature profiles of different GB loading conditions (one basket: 1, 1.5, and 2 kg) during HARF roasting (100 °C HA, 10 kW RF at a gap of 16 cm).

Figure 2 shows moisture/temperature profiles for single-basket loads (1, 1.5, 2 kg) during HARF roasting with 100 °C hot air and a 10 kW RF. All exhibited rapid internal heating: the moisture dropped quickly within the first 10 min while the temperature rose steadily beyond 120 °C, favoring browning and flavor development. The 1 kg load reached 110 °C within 6 min and ≤0.05 g/g (d.b.) by 30 min; the 1.5 kg load reached a similar moisture level by about 24 min; and the 2 kg load completed in just 22 min, reaching 145 °C—indicating that an increased load did not prolong the time needed due to greater RF absorption.

**Figure 3:**
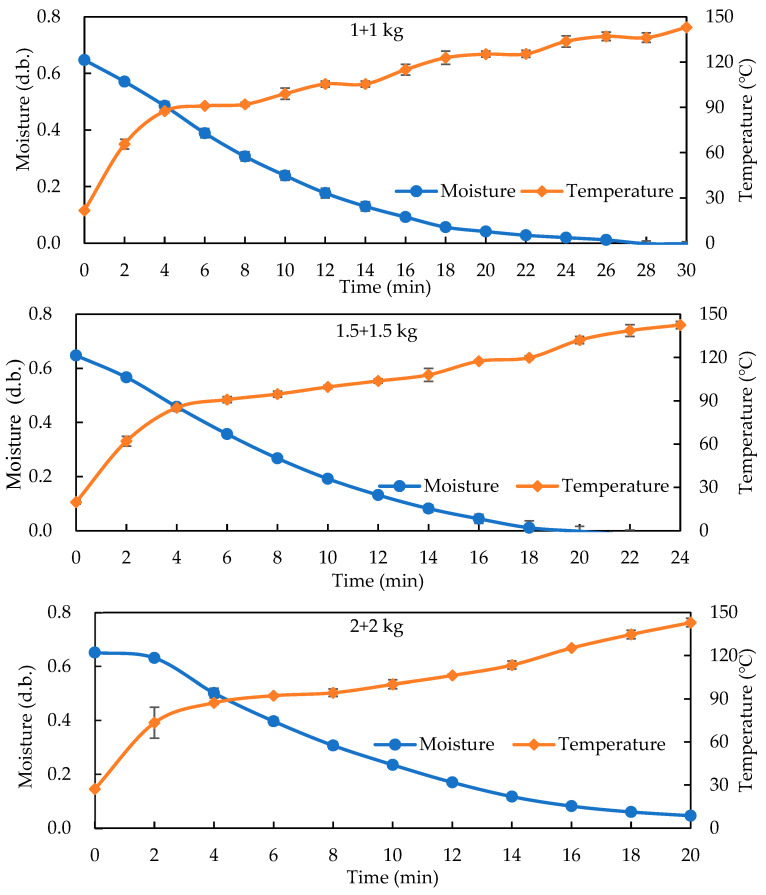
The drying curves and temperature profiles of different GB loading conditions (double baskets: 1 + 1, 1.5 + 1.5, and 2 + 2 kg) during HARF roasting (100 °C HA, 10 kW RF at a gap of 16 cm).

Figure 3 shows analogous behavior for double-basket loads (1 + 1, 1.5 + 1.5, and 2 + 2 kg), which required approximately 30, 24, and 20 min, respectively, to reach temperatures above 140 °C. The moisture content decreased sharply within the first 6 min due to rapid internal dielectric heating, followed by a slower reduction as surface evaporation became dominant. The 2 + 2 kg treatment showed the fastest drying and most stable temperature rise, indicating that a higher load improved energy absorption and reduced reflection losses between electrodes.

The double-basket configuration also produced smoother temperature and moisture gradients than single-basket roasting, suggesting more uniform volumetric heating and improved thermal efficiency. These results indicate that a proper load distribution within the electrode field not only accelerates drying but also minimizes localized overheating, providing a more stable and controllable roasting process. Overall, the 2 + 2 kg setting at a 16 cm gap achieved the best balance between heating rate, energy efficiency, and product stability, making it suitable for future continuous HARF processing.

**Figure 4:**
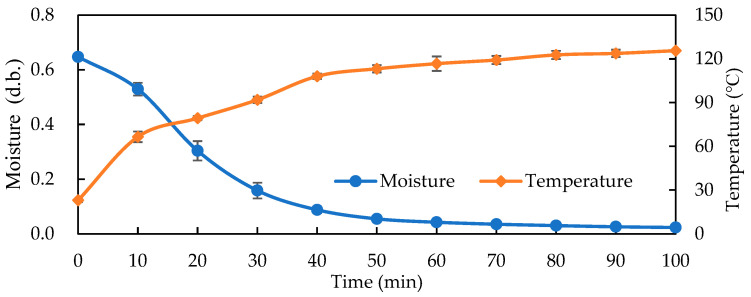
Drying curves and temperature profiles of different GB during 140 °C hot air oven roasting.

Figure 4 depicts cyclone oven drying at 140 °C: moisture content (d.b.) decreased from about 0.66 g/g to 0.15 g/g within 30 min and approached 0.05 g/g by 60 min; temperature rose from 25 °C to 2 °C by about 50 min, stabilizing at 125~130 °C. Compared with HARF processing (Figure 2 and Figure 3), conventional hot air required over triple the time for similar terminal temperatures, due to surface-limited heat transfer [13,14]. In contrast, 10 kW HARF completed the direct roasting of wet GB into GB tea within 20~30 min. In contrast, hot air, though stable, heated more slowly with flatter drying curves and potential internal/external temperature gradients impacting quality.

Li et al. [1] observed that hot air-only (140 °C) drying for germinated wheat required ≥ 18 min with about 8.7 kWh/kg energy consumption; adding RF at the same temperature shortened the time to <10 min and better preserved phenolics/antioxidants. Chitsuthipakorn et al. [15] reported that 15 kW, 27.12 MHz RF + 55 °C hot air required ~330 min to reduce paddy from 25 to 26% to <14% moisture, which is still far less efficient than the present results.

Table 1 compares the energy/time for HARF vs. a cyclone oven at different loads during direct roasting to 5% moisture. HARF processing greatly shortened the total time versus the oven’s 100 min. For single baskets (1, 1.5, and 2 kg), HARF processing required 30, 24, and 22 min with a total energy about 3.19, 2.82, and 2.92 kWh/kg, respectively—indicating improved energy efficiency at higher loads. By contrast, the oven’s combined drying/roasting consumed ~15.19 kWh/kg over 100 min.

Double-basket treatments showed similar trends: 1 + 1, 1.5 + 1.5, and 2 + 2 kg required 30, 24, and 20 min with ~3.68, 3.54, and 3.48 kWh/kg, respectively. Notably, in a continuous-processing context, HARF processing at 2 + 2 kg required only 0.87 kWh per kg of GB tea—a compelling basis for scale-up. Overall, 20~30 min complete drying/roasting at high power with appropriate loading dramatically increased rates and reduced energy, supporting pilot-to-industrial continuous production with a high efficiency, low energy, reduced cost, and stable quality.

### 3.2. Sensory Evaluation and Aroma Analysis of GB Tea

Table 2 shows significant color changes in GB post-processing. RF (10 kW HARF, 140 °C) exhibited L* = 18.57, a* = 3.51, b* = 6.12, and ΔE = 7.44, the smallest color difference versus the control (N (untreated): L* 25.99, a* 3.04, and b* 5.77) and cyclone oven roasting (OV: L* = 22.96, a* = 4.18, b* = 7.88, and ΔE = 3.86), indicating the better preservation of intrinsic buckwheat color. Commercial buckwheat seeds (L* = 34.21, a* = 3.04, b* = 16.41, and ΔE = 13.45) were brightest/most browned, likely due to different processing or unhulled buckwheat.

Xu et al. [16] noted that rapid RF heating with short holds reduces browning and stabilizes color; Schlörmann et al. [17] found that 140 °C roasting can induce a moderate Maillard reaction while curbing acrylamide. Thus, HARF processing avoids over-browning (and associated bitterness) while preserving the natural cereal hues that consumers prefer, enhancing visual appeal and competitiveness. Li et al. [1] reported that the RF drying of germinated cereals lowered heat loss and preserved phenolics/antioxidants, improving sensory quality. Zhang et al. [3] found that HARF drying preserved the aroma in germinated mung bean tea and increased acceptability—underscoring RF’s advantages for functional germinated teas.

Table 3 summarizes the nine-point hedonic results for GO140 (cyclone oven 140 °C), GRF140 (RF to 140 °C), and a commercial buckwheat tea. GRF140 scored highest in color (6.00), aroma (6.85), flavor (6.22), and overall liking (6.40). GO140 ranked second for color and overall, while the commercial sample scored lowest; its color/flavor was significantly inferior to GRF140 (*p* < 0.05). Hence, HARF roasting not only speeds up drying but also preserves sensory quality—especially aroma—aligning with the literature on HARF’s preservation of aroma compounds and highlighting its industrial potential over conventional hot air.

**Figure 5:**
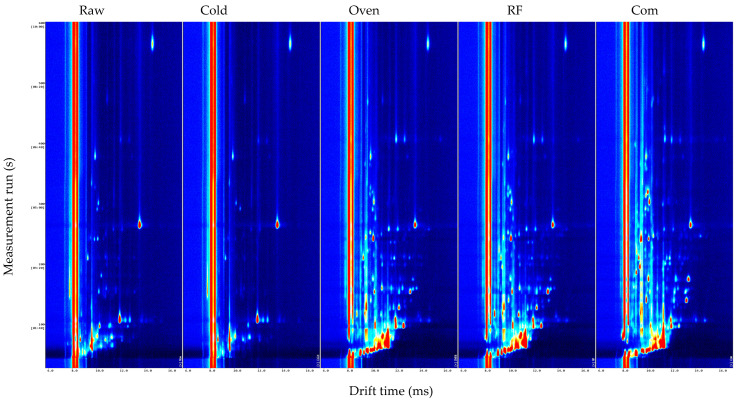
GC-IMS plot comparison of raw buckwheat, GB dried with 45 °C cold air, RF roasting (10 kW, 40.68 MHz), hot air oven roasting, and commercial buckwheat tea. 1. Raw represents raw buckwheat. Cold represents GB dried with 45 °C cold air. Oven means hot air oven roasting. Com represents commercial buckwheat tea.

Figure 5 compares the GC-IMS fingerprints across processes. Raw buckwheat exhibited fewer, weaker peaks concentrated at 1.0–2.0 ms drift times, indicating limited volatile diversity. Cold air-dried, germinated samples showed additional peaks, suggesting the germination-driven generation of precursors/low-MW volatiles, but signals remained below roasted teas. RF and oven roasting GB tea produced numerous high-intensity peaks at 1.0–2.5 ms and 6.0–14.0 ms, consistent with Maillard- and lipid oxidation-derived volatiles. RF samples showed more focused distributions and a lower background, reflecting uniform heating and a shorter treatment that may reduce over-reaction products. Commercial buckwheat tea resembled RF-roasted GB but with higher intensities at some positions—likely due to cultivar, processing, and storage differences.

Overall, roasting—especially RF—substantially increased volatile richness/intensity, improving aroma quality. Thermal treatment broadly promoted Maillard/lipid oxidation products (e.g., aldehydes, furans, pyrazines), and GC-IMS proved highly sensitive and discriminating for these changes. RF’s shorter time and reduced thermal degradation yielded clearer aroma enhancement, a relatively underexplored area offering novelty and application potential [5].

**Figure 6:**
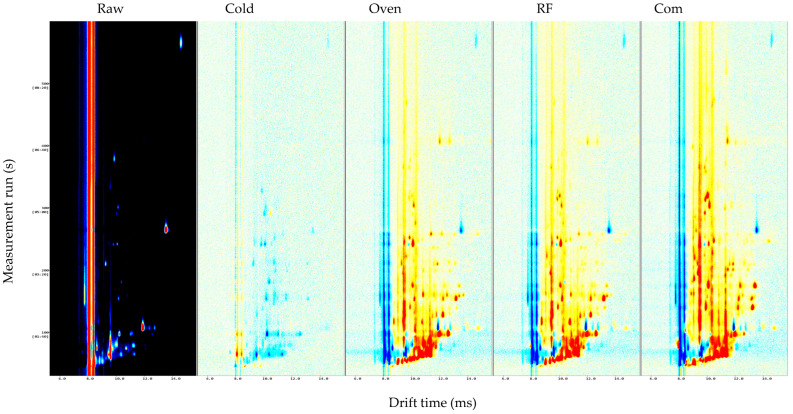
GC-IMS 3D topographic plots of raw buckwheat, GB dried with 45 °C cold air, RF roasting (10 kW, 40.68 MHz), hot air oven roasting, and commercial buckwheat tea. 1. Raw represents raw buckwheat. Cold represents GB dried with 45 °C cold air. An oven represents hot air oven roasting. Com represents commercial buckwheat tea.

The 3D plots (Figure 6) highlighted intensity differences: raw had the fewest/weakest peaks; cold air-dried increased signals at shorter drift times; and roasted GB teas displayed dense, high-intensity red/yellow peaks at longer drift times, indicating abundant roast products. The commercial sample resembled oven-roasted GB but with more dispersed peaks, likely reflecting raw material/process variability. This matches HS-GC-IMS cereal studies [18], where roasting elevates pyrazines/aldehydes/ketones and reduces alcohols/esters/sulfur species. Peng et al. [19] likewise found that germination and roasting markedly reshaped quinoa volatiles, with germination enhancing floral/fresh notes and roasting amplifying caramel/cocoa/nutty notes while reducing off-odors.

**Figure 7:**
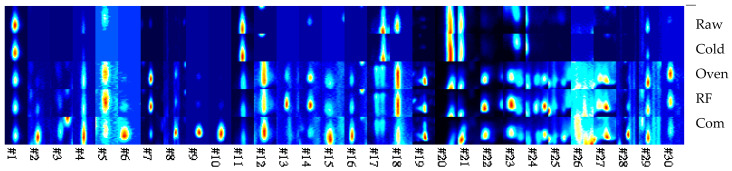
Gallery plot of raw buckwheat, GB dried with 45 °C cold air, RF roasting (10 kW, 40.68 MHz), hot air oven roasting, and commercial buckwheat tea. 1. Raw represents raw buckwheat. Cold represents GB dried with 45 °C cold air. An oven represents hot air oven roasting. Com represents commercial buckwheat tea.

Figure 7 shows that different treatments led to significant variations in the distribution of volatile compounds in buckwheat. Raw buckwheat exhibited the lowest number and intensity of peaks, while the cold air-dried germinated sample showed increased signals in the low-to-medium drift time region, reflecting enzyme activation during germination that promoted the formation of floral aroma precursors. Both cyclone oven and radio frequency roasting produced high-intensity peaks at multiple compound positions, with HARF treatment displaying the widest distribution and strongest intensities, indicating its pronounced effect in promoting the formation of Maillard reaction products (such as pyrazines, aldehydes, and ketones). The distribution pattern of commercial buckwheat tea was similar to that of the cyclone oven but with more dispersed peaks, likely due to differences in raw materials and processing conditions.

Zieliński et al. [20] reported that roasting not only affects aroma but also leads to protein quality deterioration, reduced antioxidant activity, and increased browning, which corresponds with our observation of the accumulation of Maillard reaction products under high-energy roasting (particularly RF). This trend is consistent with the findings of Fan et al. [5], who showed that when buckwheat was roasted at 180–220 °C, key aroma-active compounds such as 2-ethyl-3-methylpyrazine and 3,5-diethyl-2-methylpyrazine significantly increased, enhancing the overall aroma intensity.

**Figure 8:**
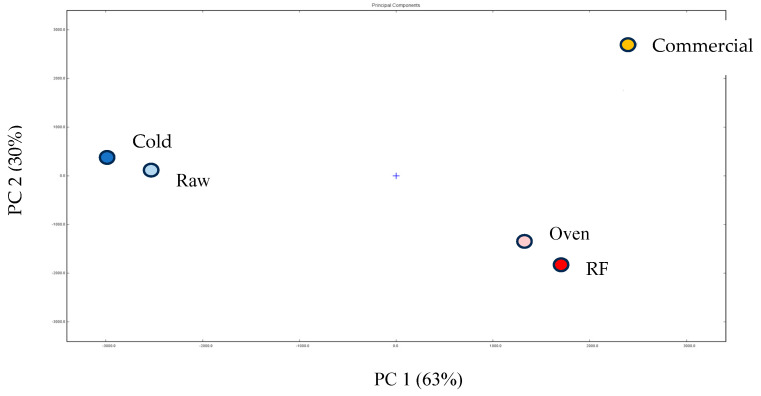
PCA of volatile compounds of raw buckwheat, GB dried with 45 °C cold air, RF roasting (10 kW, 40.68 MHz), hot air oven roasting, and commercial buckwheat tea. 1. Raw represents raw buckwheat. Cold represents GB dried with 45 °C cold air. An oven represents hot air oven roasting. Com represents commercial buckwheat tea.

The PCA (Figure 8) visualized the variance among treatments: PC1 = 63% and PC2 = 30%. Raw and cold clustered to the left (similar, fresher profiles). Oven- and RF-roasted GB teas clustered together to the lower right, clearly separated from raw/cold, reflecting shared roasted attributes rich in pyrazines/caramelization products. The commercial tea appeared at the upper right, separated from oven/RF GB teas, indicating distinct volatiles likely due to raw material or composite processing differences—consistent with Fan et al. [5], who reported roasted samples separating due to elevated pyrazines/caramelization products. Zieliński et al. [20] further attributed chemical profile shifts to protein structural changes and increased browning during heating, explaining the divergence between high-temperature roasted and unroasted groups.

## 4. Conclusions

Using 100 °C hot air and a 10 kW RF with a 16 cm electrode gap to directly roast wet GB and reduce moisture content from 40% to 5%, reaching a product temperature of 140 °C. With a single-basket 2 kg load, the energy consumption was 1.46 kWh per kg of GB tea, while double baskets (2 + 2 kg) had the lowest energy consumption of 0.87 kWh per kg, which is well suited for future continuous RF roasting. Compared with a conventional cyclone oven, HARF processing shortened the drying time to one-fifth and the per-kg energy for GB tea to one-eighteenth, dramatically improving the production efficiency and energy use. Moreover, HARF-processed products showed a superior color, flavor, and overall acceptability versus hot air and commercial teas—especially in aroma and mouthfeel—consistent with prior findings that RF reduces nutrient and flavor loss. GC-IMS and PCA confirmed that roasting (oven, RF) significantly promoted Maillard product development, with RF yielding the highest aroma intensity/complexity—supporting product development and market promotion.

## Figures and Tables

**Table 1:** Effect of processing methods on the energy consumption of GB tea.

RF Loading Capacity (kg)	Average Current (A)	* Time (min)	Total Energy Consumption (kWh)	Energy (kWh/kg)
1 kg	16.73	30	3.19	3.19
1.5 kg	18.54	24	2.82	1.88
2 kg	20.96	22	2.92	1.46
1 + 1 kg	19.27	30	3.68	1.84
1.5 +1.5 kg	23.14	24	3.54	1.18
2 + 2 kg	27.25	20	3.48	0.87
1 kg Oven	38.06	100	15.91	15.91

1. The initial moisture content was 40%. 2. * Time required to obtain 5% moisture content of GB. 3. Note: Data represent technical measurements from RF equipment performance; statistical comparison was not applicable.

**Table 2:** Color of GB tea under various processing methods.

Treatment	L *	a *	b *	Δ*E*
N	25.99 ± 2.57 ^b^	3.04 ± 0.50 ^b^	5.77 ± 1.00 ^c^	0
RF	18.57 ± 2.57 ^d^	3.51 ± 0.51 ^b^	6.12 ± 1.73 ^c^	7.44
Oven	22.96 ± 2.15 ^c^	4.18 ± 0.37 ^a^	7.88 ± 1.10 ^b^	3.86
Commercial	34.21 ± 1.98 ^a^	3.04 ± 0.50 ^a^	16.41 ± 1.22 ^a^	13.45

1. N represents untreated. RF represents 10 kW HARF 140 °C. The oven represents a 140 °C oven. 2. Data were presented as mean ± S.D. (*n* = 6). 3. Means with different superscript letters in the same column were significantly different (*p* < 0.05).

**Table 3:** Nine-point hedonic sensory evaluation of different buckwheat teas.

Item	GO140	GRF140	Commercial
Color	6.29 ± 1.56 ^a^	6.00 ± 1.67 ^b^	5.49 ± 2.07 ^bc^
Smell	5.62 ± 1.67 ^b^	6.85 ± 1.54 ^a^	5.16 ± 1.74 ^b^
Taste	5.58 ± 2.08 ^a^	6.22 ± 1.65 ^a^	5.02 ± 2.04 ^b^
Overall	6.05 ± 1.08 ^b^	6.40 ± 1.56 ^a^	5.42± 1.84 ^bc^

1. GO140 represents GB hot air oven at 140 °C. GRF140 represents GB radio frequency at 140 °C. The commercial buckwheat tea was purchased from Kuang Chuan Foods. 2. Data are presented as mean ± S.D. (*n* = 56). 3. ^a–c^ Means with different superscript letters in the same row are significantly different (*p* < 0.05).

## Data Availability

The original contributions presented in the study are included in the article/Supplementary Material, further inquiries can be directed to the corresponding author.

## Supplementary Materials

The following supporting information can be downloaded at: https://www.mdpi.com/article/10.3390/foods14213596/s1, Figure S1: Schematic diagram of the experimental workflow for the preparation and evaluation of germinated buckwheat (GB) tea.

## References

[1] Li X., Wang Y., Chen J., Zhang L., Liu H. (2021). Effects of radio frequency assisted hot air drying on germinated wheat: Drying kinetics and quality attributes. J. Food Eng..

[2] Kawatra N., Jha G., Dubey A. (2023). Effect of selected elicitors on phytochemical content and antioxidant activity of buckwheat (*Fagopyrum esculentum*) sprouts obtained from seeds cultivated using the hydroponics technology. Int. J. Food Sci. Technol..

[3] Zhang Y., Pandiselvam R., Zhu H., Su D., Wang H., Ai Z., Kothakota A., Khaneghah A.M., Liu Y. (2022). Impact of radio frequency treatment on textural properties of food products: An updated review. Trends Food Sci. Technol..

[4] Ling B., Cheng T., Wang S. (2020). Recent developments in applications of radio frequency heating for improving safety and quality of food grains and their products: A review. Crit. Rev. Food Sci. Nutr..

[5] Fan X., Zhong M., Feng L., Huo Y., Pan L. (2024). Evaluation of flavor characteristics in tartary buckwheat (*Fagopyrum tataricum*) by E-nose, GC-IMS, and HS-SPME-GC-MS: Influence of different roasting temperatures. LWT.

[6] Ma C., Nie H., Liu L.-X., Wang F.-R., Chen Y., Zhang W., Liu Y.-G. (2024). Gas chromatography–ion mobility spectrometry (GC-IMS) technique and its recent applications in grain research. J. Sci. Food Agric..

[7] Zhang B., Cao M., Wang X., Guo S., Ding Z., Kang Y., Hu L., Xiong L., Pei J., Ma Y. (2024). The combined analysis of GC-IMS and GC-MS reveals the differences in volatile flavor compounds between yak and cattle-yak meat. Foods.

[8] Parastar H., Weller P. (2024). Towards greener volatilomics: Is GC-IMS the new Swiss army knife of gas phase analysis?. TrAC Trends Anal. Chem..

[9] Tsou S.-F., Hsu H.-Y., Chen S.-D. (2024). Effects of different pretreatments on the GABA content of germinated brown rice. Appl. Sci..

[10] Castro-Alba V., Lazarte C.E., Perez-Rea D., Sandberg A.S., Carlsson N.G., Almgren A., Bergenståhl B., Granfeldt Y. (2019). Effect of fermentation and dry roasting on the nutritional quality and sensory attributes of quinoa. Food Sci. Nutr..

[11] Ryu J.Y., Choi Y., Hong K.H., Chung Y.S., Cho S.K. (2020). Effect of roasting and brewing on the antioxidant and antiproliferative activities of tartary buckwheat. Foods.

[12] Teseme W.B., Weldeselassie H.W. (2020). Review on the study of dielectric properties of food materials. Am. J. Eng. Technol. Manag..

[13] Zhou X., Wang S. (2019). Recent developments in radio frequency drying of food and agricultural products: A review. Dry. Technol..

[14] Chen Y.-H., Yen Y.-F., Chen S.-D. (2021). Effects of radio frequency heating on the stability and antioxidant properties of rice bran. Foods.

[15] Chitsuthipakorn K., Thanapornpoonpong S.N. (2021). Quality of milled rice from large-scale dried paddy rice by hot air combined with radio frequency heating. Processes.

[16] Xu J., Yang G., Li R., Xu Y., Lin B., Wang S. (2022). Effects of radio frequency heating on microbial populations and physicochemical properties of buckwheat. Int. J. Food Microbiol..

[17] Schlörmann W., Zetzmann S., Wiege B., Haase N.U., Greiling A., Lorkowski S., Dawczynski C., Glei M. (2020). Impact of different roasting conditions on sensory properties and health-related compounds of oat products. Food Chem..

[18] Wang C., Zhang M., Mujumdar A.S., Sun J. (2022). Characterization of volatile compounds in roasted grains by HS-GC-IMS. Foods.

[19] Peng S., Li Y., Yang X., Liu X. (2024). Influence of germination and roasting on the characteristic aroma of quinoa: HS-GC-IMS and HS-SPME-GC-MS analysis. J. Food Compos. Anal..

[20] Zieliński H., Michalska A., Piskuła M.K., Kozłowska H. (2009). Changes in protein quality and antioxidant properties of buckwheat seeds and groats induced by roasting. J. Agric. Food Chem..

