# Supplementary material for: Enhancing Quality and Processing Efficiency of Germinated Buckwheat Tea Through Hot Air-Assisted Radio Frequency Roasting
Source: Foods. 2025 Oct 22;14(21):3596. doi: 10.3390/foods14213596 (PMC12607411; doi:10.3390/foods14213596)
Supplement: Supplementary file 1 [file foods-14-03596-s001.zip › foods-3902380-supplementary.pdf]

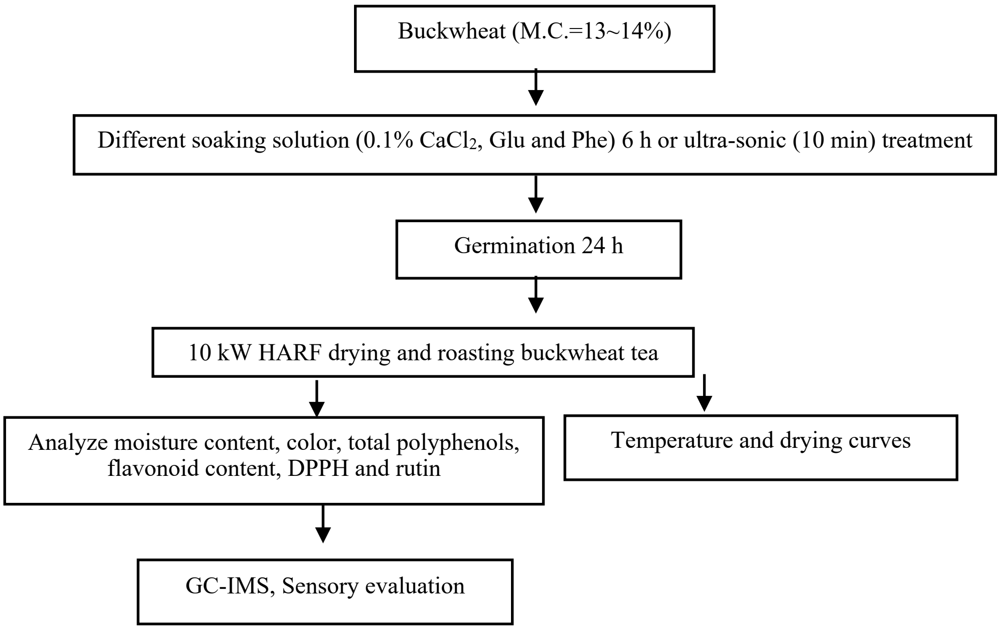

Figure S1. Schematic diagram of the experimental workflow for the preparation and evaluation of germinated buckwheat (GB) tea. The process includes soaking (25 °C, 6 h), germination (25 °C, 24 h, dark), and hot air-assisted radio frequency (HARF) roasting (10 kW, 40.68 MHz, 100 °C, forced air 2.5 m/s). After roasting, color analysis, 9-point hedonic sensory evaluation, and GC–IMS aroma profiling were performed to assess the physicochemical and sensory quality of GB tea. This workflow illustrates the sequence and relationships among each processing and analytical step described in the study.
